# Case Series: Fibula Free Flap with Bone Allograft as the Gold Standard in Lower Limb-Salvage Surgery for Adolescent Patients with Primary Bone Tumors Located within Tibial Diaphysis: Technical Modifications and Short-Term Follow-Up †

**DOI:** 10.3390/jcm13144217

**Published:** 2024-07-19

**Authors:** Jakub Opyrchał, Daniel Bula, Krzysztof Dowgierd, Bartosz Pachuta, Dominika Krakowczyk, Anna Raciborska, Łukasz Krakowczyk

**Affiliations:** 1 Department of Oncology and Surgical Oncology for Children and Youth, Institute of Mother and Child, 01-211 Warsaw, Poland; 2 1st Department of Oncologic Surgery, Maria Sklodowska Curie Memorial National Cancer Center, 44-100 Gliwice, Poland; 3 Department of Clinical Pediatrics, Head and Neck Surgery Clinic for Children and Young Adults, University of Warmia and Mazury, 10-709 Olsztyn, Poland; 4 Pediatric Surgery and Urological Department, Upper Silesian Child Health Center in Katowice, Silesian University of Medicine, 40-052 Katowice, Poland

**Keywords:** osteosarcoma, limb-salvage surgery, bone tumors, microsurgery, fibula free flap, case series

## Abstract

**Background:** Primary malignant bone tumors are most commonly associated with mutilating surgical procedures that can significantly disturb the motor development of a young patient and are frequently affiliated with major postoperative complications. Unfortunately, despite available autologous tissue donor sites, artificial materials are still most commonly used for the reconstruction of post-resection defects. Reconstructive microsurgery is increasingly recognized as an effective method of functional reconstruction, creating the possibility of performing limb-sparing surgery (LSS) with significant limitation of major postoperative complications at the same time. **Methods:** The study group consisted of 9 pediatric patients diagnosed with primary malignant bone tumor in the limb location. In order to perform microvascular reconstruction, 9 free fibula flaps were used in combination with a bone allograft (Capanna method). The functional outcome of the reconstruction was assessed on the basis of the MSTS (Musculoskeletal Tumor Society Scoring System) scale. **Results:** The presented analysis proves the effectiveness of this reconstructive procedure and the possibility of performing LSS with reasonable functional outcomes after appropriate patient qualification. In this study, all limbs included were spared. In all cases, the R0 surgical margins were achieved and no reports of local recurrences were reported during the follow-up. The average score on the MSTS scale was 27/30 points. **Conclusions**: Microvascular reconstructive surgery is an individually personalized and highly effective method of treating patients with primary bone tumors in the limb location and provides satisfactory functional outcomes.

## 1. Introduction

Year by year, the effectiveness of reconstructive treatment of post-resection defects in pediatric patients is becoming the subject of more and more heated discussions. In some clinical situations, there is still a lack of studies on the appropriate number of cases to be able not only to draw appropriate conclusions, but also to try to establish treatment guidelines. Of all childhood neoplasms, bone tumors seem to be the most dangerous, with an ability to create indisputable disorders in developmental age. Osteosarcoma and Ewing sarcoma are the two most common malignant bone tumors in the group of pediatric patients. These types of neoplasms require surgical management as a part of the multidisciplinary approach. At initial diagnosis, around 20% of patients present with dissemination of cancer cells to lungs, whereas 40% of patients develop metastases at a later stage [1].

Due to the relative rarity of these tumors, the optimal surgical treatment for pediatric patients with osteosarcoma has not been fully established and till this day is a matter of debate. Unfortunately, despite available autologous tissue donor sites, artificial materials are still most commonly used for the reconstruction of post-resection defects. With advances in neoadjuvant and adjuvant chemotherapy and a better understanding of the biology of bone and soft-tissue sarcomas, limb-salvage surgery with the use of reconstructive microsurgery has become a more common clinical management, striving to spare the limb while obtaining the best possible functional outcome, which is especially important in younger patients. Nowadays, more than 70% of patients with localized osteosarcoma of an extremity undergo limb-salvage surgery, but still some authors report that amputation can reduce the risk of possible complications and avoid growth-related problems associated with limb salvage [2]. Different studies suggest that the survival rate of patients largely depends on the margins of excision and the responsiveness of the primary tumor to chemotherapy. It is reported by Picci et al. that among patients with a narrow margin of excision and less than 90% necrosis of the tumor after neoadjuvant chemotherapy, local recurrence can be as high as 30% [3]. Thus, a very controversial question arises, which is whether the high-risk group should be qualified for immediate amputation to prevent local recurrence and improve their chance of survival. As mentioned before, functional limb-salvage following radical resection (R0) has become the gold standard in treating this type of neoplasm. Microsurgical reconstructions with the use of free flaps in pediatric patients has gained widespread acceptance after an initial period of concern regarding the technical possibility of the procedure as well as major complications, like postoperative fractures in particular. 

**Figure 1:**
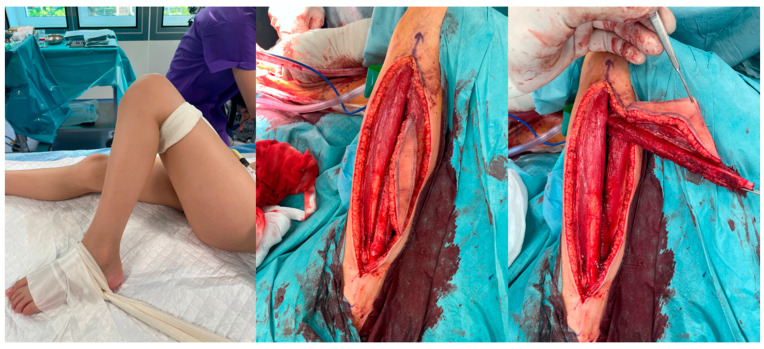
Intraoperative photographs of free osteocutaneous fibula flap harvest. Skin paddle is indicated by surgical forceps.

Because of implementing techniques such as supermicrosurgery and with advances in microsurgical materials (smaller sutures as well as surgical needles), microsurgical free flap transfer in the pediatric age group is no longer as challenging as it once was and should be always considered as the first choice of reconstructive treatment after appropriate patient qualification. Complex bone and soft tissue restoration using reconstructive microsurgery can also widen the group of patients with advanced sarcomas, previously considered inoperable, because it can provide the possibility to resect more tissue (to achieve R0 radical resection) without worrying about wound closure or functional outcome. Furthermore, these types of procedures have the great advantage of closing the wound with vascularized tissue and the use of a distant donor site that does not alter the function of an already compromised limb—which occurred and was frequently the cause of serious complications after the use of older and less sophisticated techniques. The choice of a specific bone flap will depend mainly on the size of the defect; e.g., for larger defects (>8 cm), the preferred method of reconstruction is a fibula free flap (FFF) presented in the Figure 1. 

**Figure 2:**
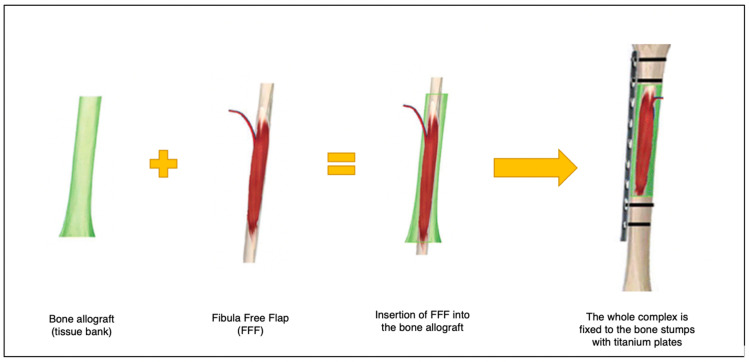
Scheme of the Capanna method. Obtaining a bone allograft from a tissue bank and taking an autograft (free fibula flap) with subsequent combination of both elements. Finally, the whole complex restores the post-resection bone defect in the tibia by fixing it to the bone stumps with titanium locking compression plates.

The bone quality is of a paramount importance for patients receiving a treatment protocol that includes adjuvant irradiation and chemotherapy, which can cause the deterioration of bone structure [4]. Another important advantage of FFFs is the fact that the length is almost always sufficient to reconstruct large defects, and in combination with bone allograft (Capanna technique) it can provide the best weight-bearing resistance of any free flaps [5] (Figure 2). Additionally, in case of bone defects with a shape that can be compared to a square (equally long and wide), the fibula flap can be used in the double barrel technique, essentially folding the bone fragment in half while doubling its thickness [6]. 

The design of this study aims to shed a new light on the results of modern limb-salvage treatment of adolescent patients. The aim of this analysis is to demonstrate the utility and usability of the FFF in combination with bone allograft and applied technical modifications in the pediatric reconstructive procedures of the lower extremities after limb-sparing sarcoma resections, as well as to report on the incidence of complications and functional outcomes.

The aspect that distinguishes this study from other scientific papers is the fact that most reports on limb reconstruction include patients with varying age groups, anatomic locations, type of reconstruction and underlaying diseases or need for surgery, whereas this study has limited the study group to adolescent patients diagnosed with primary bone tumors treated with the use of a microvascular fibula free flap in combination with bone allograft as a part of surgical management.

## 2. Materials and Methods

**Figure 3:**
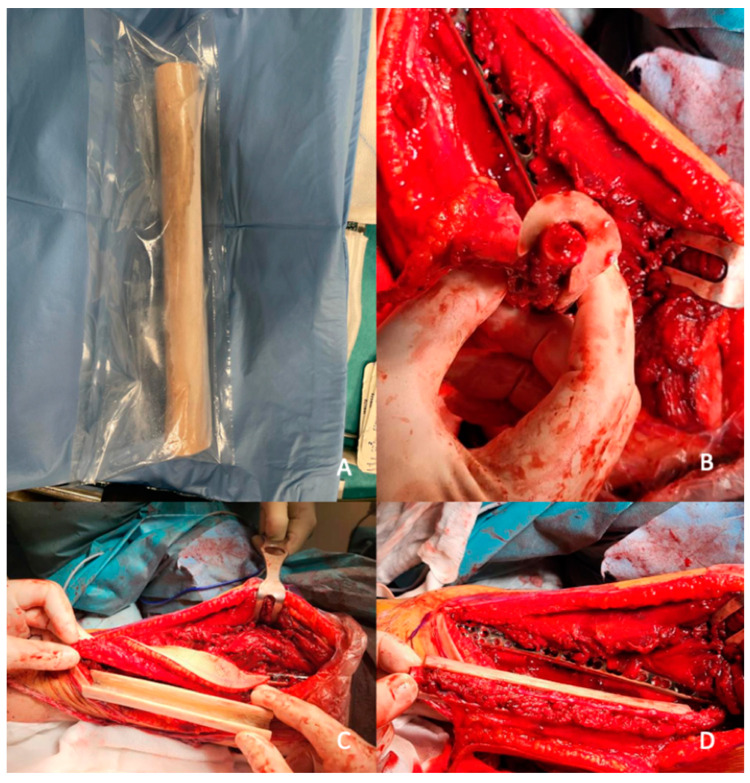
Intraoperative photographs presenting the Capanna technique. (**A**) Bone allograft obtained from a tissue bank; (**B**–**D**) insertion of a free fibula flap inside the bone allograft.

This case series study presents an analysis of 10 consecutive pediatric patients who underwent extensive oncologic resection of a primary bone tumor within the lower limb and subsequent reconstruction of the defect using microsurgical procedures; i.e., fibula free flap combined with bone allograft (Capanna technique) [7]. All procedures were performed at the Institute of Mother and Child by the same team of surgeons. The scheme of this method as well as intraoperative photographs are presented below (Figure 2 and Figure 3).

Inclusion criteria were as follows: primary malignant bone tumors sensitive to neoadjuvant chemotherapy; diaphysis tibial bone tumors not infiltrating major blood vessels and nerves; appliance of Capanna technique as a reconstructive method.

All patients with tibial primary bone tumors extensively involving the epiphysis, poor tumor response to neoadjuvant chemotherapy or incomplete follow-up were excluded from this study.

Patient data collected included demographics, age, gender, histology of the tumor, adjuvant oncologic treatment, TNM staging, type of bone involved, length of the defect, surgical margin and type of flap used for reconstruction as well as presence of local recurrence or metastases during the follow-up. 

Patients were followed up at 3 month intervals for up to 5 years postoperatively. Check-ups included clinical examination and plain radiographs of the treated extremity for the assessment of bone union, as well as exclusion of asymptomatic fractures or pseudoarthrosis.

To objectively assess the functional outcomes, each patient was evaluated using the Musculoskeletal Tumor Society Rating Scale (MSTS), which is the most thoroughly validated questionnaire in orthopedic oncology [8]. The lower extremity subscale comprises six items: pain, function, emotional acceptance, need for external support, walking ability and gait alteration. Each item is rated on a scale from 0 to 5, with a total score ranging from 0 to 30. Higher scores indicate better function. The MSTS assessment was completed for each patient 18 months after the surgical procedure. 

The functional outcome was further evaluated by considering the immobilization period and the time to achieve partial and full weight-bearing. Weight-bearing status was assessed and recorded at each follow-up visit, categorized into three levels: non-weight bearing, partial weight-bearing (requiring external support for ambulation) and full weight-bearing (movement without any external aid). Additionally, all cases were analyzed for complications during the early postoperative period, including infection, microvascular complications, wound dehiscence and seroma or hematoma formation.

As part of their oncologic follow-up, patients also had chest X-rays and computed tomography (CT) scans every 6 months to rule out any recurrence of disease or metastasis for the first 2 years after surgical treatment.

This case series has been reported in line with the PROCESS Guidelines.

## 3. Results

### 3.1. Demographics and Pathology

Altogether, there were 9 pediatric patients that underwent resection of long bone sarcomas with subsequent microsurgical reconstruction in our department. The mean age of our patients was 13.4 years (range 11–16 years). There were 3 females (33%) and 6 males (67%). The most common histopathologic diagnosis was osteosarcoma in 6 patients (67%) followed by Ewing’s sarcoma in 3 patients (33%). Tumors involved tibial epiphysis in all (100%) cases. Group characteristics are presented in Table 1.

According to the tumor grade, 6 cases were classified as low-grade (G1) and 3 cases as high-grade tumors (G2–G3). Referring to the AJC TNM Staging System for Bone Cancers, 4 patients were classified as stage IA, 2 patients as stage IB, 2 patients as stage IIB and 1 patient as stage IVA.

### 3.2. Treatment

All patients in our study group received induction chemotherapy. The mean length of the bone defect resulting from oncologic resection was 12.4 cm (range 8–16 cm). All patients (100%) had negative surgical margins after wide resection, classified as R0—indicating no cancer cells were seen microscopically at the primary tumor site.

In every case, a combination of fibula free flap (FFF) with bone allograft, also known as the Capanna technique, was applied. Intraoperative photographs of the fibula free flap harvest are presented in Figure 1. All flaps were harvested as osteocutaneous flaps, including a skin paddle to facilitate postoperative clinical monitoring of fibula graft viability and to eliminate the need for imaging studies to assess the blood supply to the fully buried bony part of the flap.

**Figure 4:**
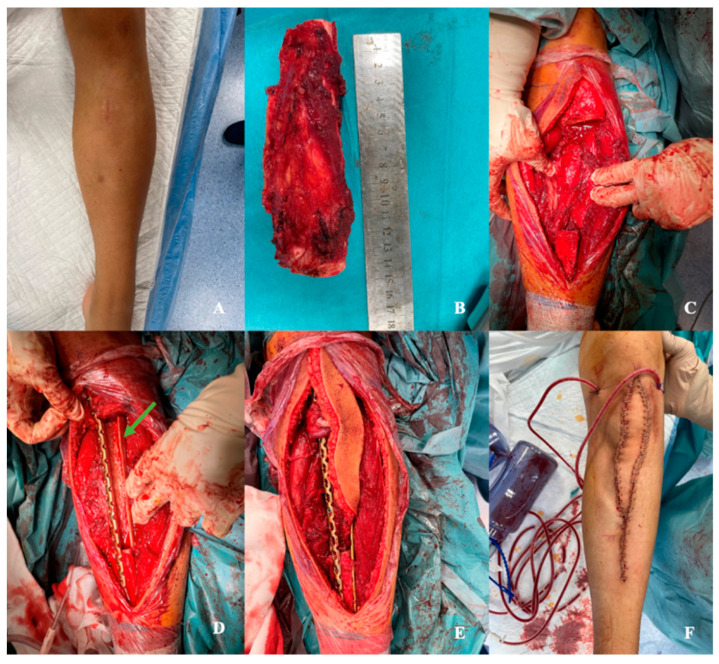
Intraoperative photos of a 14-year-old patient diagnosed with osteosarcoma of the left tibia, whose defect was reconstructed using the Capanna technique. (**A**) Preoperative photo of the left lower leg, visible scar from a surgical open tumor biopsy. (**B**) Resected specimen—more than 16 cm long together with adjacent tissues and the biopsy canal. (**C**) Extensive post-resection defect within the lower leg, visible stumps of the tibial bone. (**D**) Inset of the anastomotic plate and bone allograft (green arrow) into the bed of the resected tumor. (**E**) Implementation of a free fibula flap with a visible skin island into the bone allograft. (**F**) Postoperative photograph with a visible skin island as a ‘monitor of viability’ of the buried parts of the flap.

**Figure 5:**
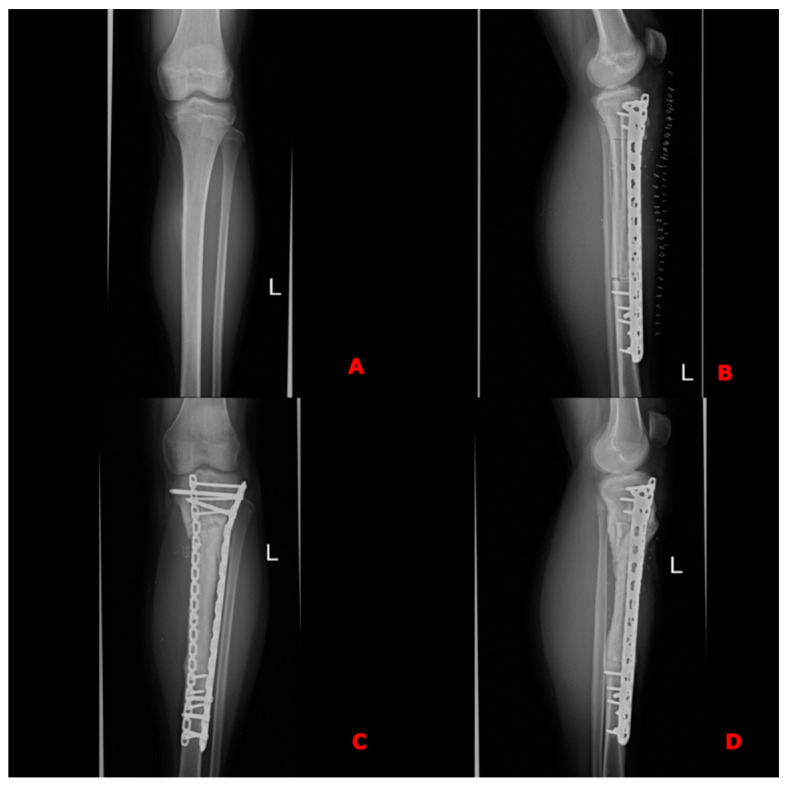
X-rays of the case shown in the figure above. (**A**) Preoperative X-ray of histopathologically confirmed osteosarcoma of the left tibia; (**B**) X-ray taken on the 1st postoperative day after resection of a specimen and receonstruction of the defect using the Cappana method, confirming the proper axis of the reconstructed bone, bony anastomoses and correct positioning of the free flap. (**C**,**D**) Follow-up X-rays in the 15th postoperative month with visible bone union and restored proper axis of the lower limb.

Since the tumors were located within the shafts of the long bones, with no involvement of the epiphyses in any case, reconstructions were performed using long diaphyseal intercalary grafts. The allograft combined with the fibula free flap was secured to the proximal and distal stumps of the tibia using locking compression plates (Figure 4D,E and Figure 5). These plates were used to facilitate earlier weight-bearing ambulation.

**Figure 6:**
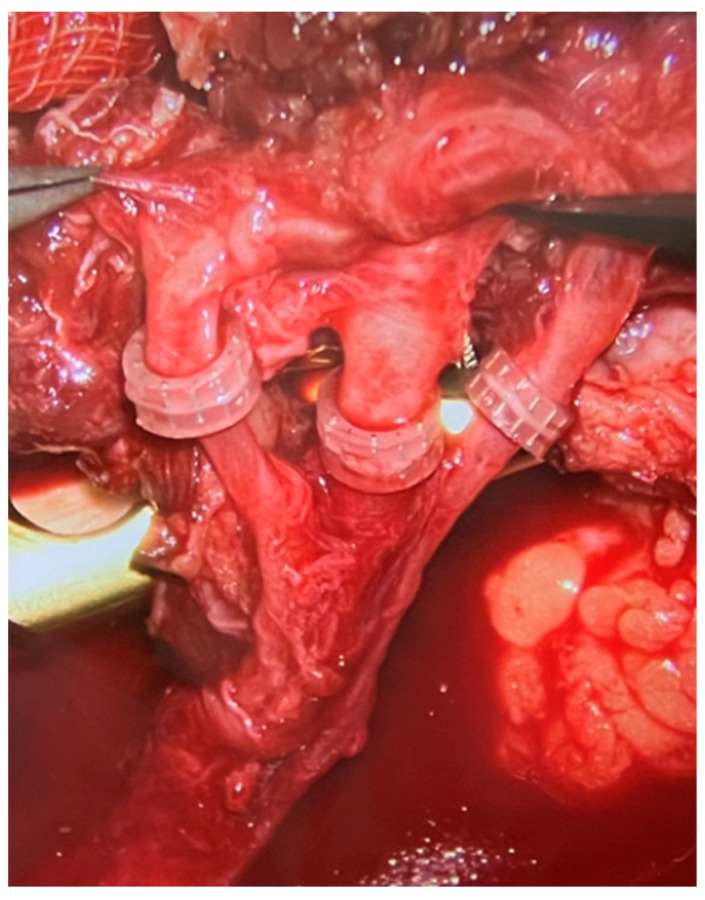
Intraoperative photograph from a surgical microscope. Three microvascular anastomoses are visible—one arterial (in the middle) and two venous (on the sides), all performed with the use of a coupler device.

The microvascular anastomoses were performed according to the available local vessels. Most commonly, the anastomoses were performed in end-to-end fashion to anterior tibial vessels. What needs to be emphasized is that in every case we performed two venous anastomoses—one to the deep venous system and one to the superficial venous system (most commonly the saphenous vein). Almost all arterial anastomoses were hand sewn using 9–0 or 10–0 nylon sutures. On the other hand, all veins were anastomosed using the microvascular anastomotic coupler system, as presented in the Figure 6 (Synovis Micro Companies Alliance, Birmingham, AL, USA). The average size of coupler used was 2.5 mm (range 1.5–4 millimeters).

Due to the small dimensions of the harvested skin paddles, with average width of 3 cm (range 2.5–4 cm), all donor sites were closed primarily without any major complications within this region reported during follow-up.

### 3.3. Flap Survival and Surgical Site Occurences

In our study group, we reported no total flap losses. There was one major early postoperative complication related to venous congestion of the skin paddle. After re-exploring the surgical site, the blood supply to the bone was palpated and confirmed using a hand-held Doppler. The skin paddle was removed due to partial loss of the cutaneous tissue, preventing possible infection from necrotic tissues. This likely resulted from significant postoperative swelling of the lower leg, which led to the compression of the perforator supplying the skin paddle and impaired its perfusion. Additionally, one minor early postoperative complication occurred as wound dehiscence on the fourth postoperative day, which was treated with simple surgical resuturing. These data are also summarized in Table 1 above.

### 3.4. Follow-Up (Functional Outcomes and Bone Union)

The mean follow-up period was 30.8 months (range 23–51 months, SD 7.9). During follow-up visits, imaging exams performed as part of oncological screening showed the presence of dissemination of the cancer in 2 (20%) patients—distant metastasis to the ipsilateral iliac bone and lung metastasis. Nevertheless, there were no cancer-related deaths reported in this study group at the time of final editorial corrections of this article.

All limbs (100%) included in this study group were spared. Patients were followed up with plain X-rays intervals of 2–3 months. On imaging studies, all microvascular fibula free flaps had developed a bony union at both ends.

**Figure 7:**
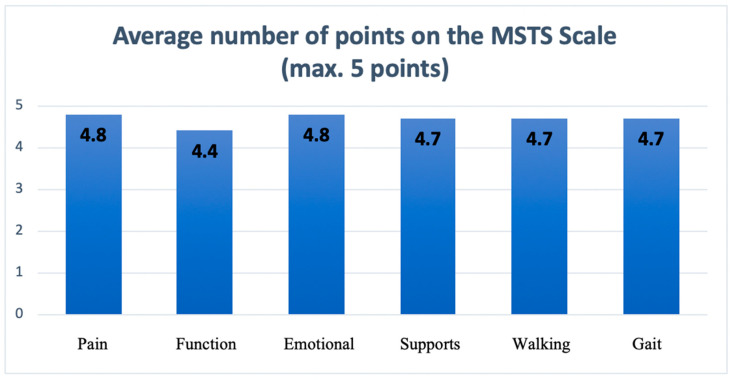
The average number of points on the MSTS scale regarding each assessed features.

Patients included in the study were also evaluated by the surgical team based on the functional MSTS scale. The mean MSTS score was 27.2 points (range 23–30 points) (Figure 7). The lowest result was reported in the patient with a necrosis of the skin island postoperatively. 

For this study group, a relatively unified protocol was established for loading the lower limb, with possible modifications based on local conditions, the length of the bone anastomosis, the length of the remaining bone stumps and the initial assessment of the anastomosis stability. Patients were allowed to bear 25% of their body weight on the treated lower limb on the third postoperative day, then 50% within 3 months of surgery, and finally, they began full weight-bearing after 12 months postoperatively. The degree of load on the limb during mobilization was controlled by a qualified team of both physicians and physiotherapists, who determined the load force on the operated limb and assessed whether a patient was ready to increase the load. The majority of patients achieved the intended goal of full weight-bearing at one year postoperatively (range 10–14 months).

## 4. Discussion

Malignant tumors of soft tissues and bones (most commonly sarcomas) in pediatric patients and young adults are most commonly associated with mutilating surgical procedures that can significantly disturb the motor development of a young patient and are often affiliated with major postoperative complications. Unfortunately, despite available autologous tissue donor sites, artificial materials are still most commonly used for the reconstruction of post-resection defects. Reconstructive microsurgery is increasingly recognized as an effective method of functional reconstruction, creating the possibility of performing limb-sparing surgery (LSS) with limitation of major postoperative complications (including those specific to artificial materials) at the same time.

Fortunately, with the development of reconstructive microsurgery in the limb region and the emergence of the field of medicine called “orthoplasty”, the prognosis and chances for full recovery for patients with advanced sarcomas of the lower limbs are full of optimism. The aim of this study is to present the results of limb-sparing treatment of advanced tibial sarcomas in adolescent patients, using a modified fibular free flap, and to present that in such cases, taking that one extra step further may give a chance for a good quality of life. Our results show that the fibula free flap combined with bone allograft can serve as a support for the entire body, as evidenced by the lack of postoperative fractures of the graft. These results should be treated very carefully because they refer to a relatively small group of patients. However, the modifications to the surgical technique as well as the postoperative care that have been introduced may help other centers achieve better postoperative functional outcomes.

With the progress and development of neoadjuvant and adjuvant treatment (i.e., chemotherapy), and at the same time a better understanding of the biology of bone and soft tissue sarcomas, limb-sparing procedures using reconstructive microsurgery techniques have become an increasingly common solution. The main aim of these procedures is to spare the limb while obtaining the best possible functional outcome, which is of paramount importance in younger patients during the period of the most dynamic motor development. Currently, worldwide, more than 70% of patients diagnosed with osteosarcoma of the limb undergo limb-sparing procedures, although the percentage of biological reconstructions still remains insignificant, in favor of the widespread use of artificial materials—endoprostheses and cement spacers [2].

In the case of primary bone tumors, functional reconstruction of the limb after radical resection has become possible using many surgical and microsurgical methods. Some of them are gradually falling out of clinical use due to numerous limitations (e.g., massive bone allografts or rotational plasties), while others are relatively modern and provide the best possible functional results, which seem to be becoming, in appropriately selected cases, the ‘’gold standard’’. One such method is microsurgical reconstruction using free flaps. This paradigm shift in the field of orthoplastic surgery happened because extendible endoprostheses, even though less morbid than biologic constructs, are reported to have a higher rate of infections and failure of lengthening mechanisms [9].

Unfortunately, due to the high complexity of microsurgical procedures involving reconstruction of post-oncological bone defects using free flaps, the overall rate of postoperative complications in certain groups of patients, available in the literature, is as high as 38–80% [10,11,12,13,14].

When it comes to reconstruction of post-resection long bone defects, the most frequently discussed weakness of this procedure is postoperative fractures. This is due to the loads to which the reconstructed areas are subjected and the resulting need for appropriate mechanical resistance. Postoperative fracture is a complication of paramount importance because it can remarkably impair and delay the physical rehabilitation of young patients, significantly limiting the patient’s activity until bone union is achieved.

The most common early complications that occur in postoperative period of microsurgical procedure are wound healing disorders, impaired flap perfusion and temporary paralysis of the superficial peroneal nerve (in the case of reconstruction with a fibula free flap) [15]. As for late complications, postoperative fractures (34%) and bone nonunion (11%) predominate in this group [11]. In the analysis conducted by Ruiz-Moya et al. on a group of 27 pediatric patients who underwent microsurgical reconstructions, as many as 14 (52%) of them required revision surgical intervention.

On the other hand, in the case of reconstruction with artificial materials (such as endoprostheses), the most common problems described in the literature seem to be periprosthetic infections, aseptic or septic loosening of the implant or malfunction of the mechanical elements of the endoprosthesis itself. Savidou et al. and Schinhan et al. also pointed out that complications related to “soft tissues” (such as limited limb mobility, wound dehiscence or infection of the surgical site) are the most common in this group of patients, with rates of 43% and 46%, respectively [16,17].

In a representative study by Savidou et al. on a group of 633 pediatric patients with endoprostheses, as many as 117 (18.5%) of them presented deep periprosthetic infection, and 102 (16%) of them had aseptic implant loosening. Both complications were most often associated with the need for revision surgical procedure and removal or replacement of the endoprosthesis. This is a type of complication that can be largely eliminated by application of free microsurgical flaps (i.e., biological reconstructions).

The low rate of complications in this study is most likely due to the relatively small size of the study group compared to the meta-analyses mentioned above, as well as technical modifications of the procedure itself and the extensive experience of the multidisciplinary team. 

From the analysis and comparison of reconstructive procedures using artificial elements and free flaps, it is evident that when vascular complications associated with the learning curve of micro-anastomosis reconstruction are eliminated, the remaining complications are strictly related to impaired healing of bone fragments. The number of these complications is very similar to those occurring in procedures without the use of microsurgery. Therefore, despite the higher level of technical complexity, reconstructive procedures using free flaps should not be dismissed for this reason.

It is also important to remember that in pediatric patients, we deal with smaller caliber vessels than in adults, but these vessels are usually healthy, without atherosclerotic changes. In adults, the presence of such changes is a primary reason for impaired blood supply to flaps in microvascular reconstructions. Comparing the degree of complications in pediatric patients is challenging due to the low number of published studies, as previously mentioned.

However, if we consider factors such as a highly qualified and experienced surgical team, modern anastomotic technologies like vascular couplers, relatively healthy recipient vessels, and effective antithrombotic therapy, it becomes clear that microsurgical procedures can potentially achieve greater effectiveness and a very low complication rate. This is not only in comparison to procedures without the use of free flaps but also to reconstructive procedures in adult patients. This may explain the low rate of complications observed in this study group.

The most significant modifications in surgical technique, both at the resection and reconstruction stages, were bone fixation technique, inclusion of flap’s skin paddle as a monitor of proper bone perfusion and double microvascular venous anastomoses of the free flap. Those modifications are described in more detail below.

### 4.1. Bone Fixation Technique

**Figure 8:**
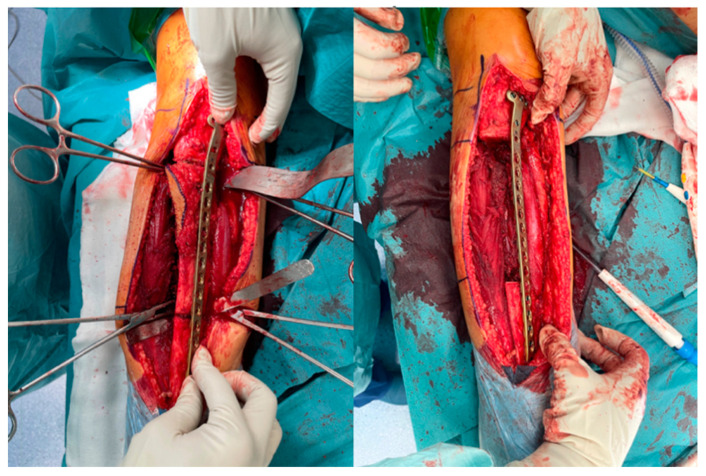
An example of fixation using the self-locking titanium plates performed before resection of the whole specimen, maintaining the primary limb axis and distance between the bone stumps.

In order to mitigate the risk of postoperative fractures, fixation using the self-locking titanium plates was performed in most cases before tumor resection. In doing so, the primary length of the reconstructed limb as well as the proper axis of the limb were preserved. Those two factors significantly reduce the risk of developing pseudoarthrosis and prevent disturbances in bone union (Figure 8).

If, in turn, titanium plates would be fixated after resection, the surgeon has to estimate the appropriate positioning of the reconstruction to correlate with the healthy limb. An important part of this technique is the fixation of plates with screws at a sufficient distance from the planned osteotomy lines. By taking this into account, if there are cancer cells in the intraoperative histopathological examination taken from the bone marrow, it is possible to widen the bone margin without the need to dismantle the anastomotic plates and thus lose the appropriate limb axis as well as the distance between bone stumps (Figure 8).

### 4.2. Skin Paddle as a Monitor of Proper Bone Perfusion

In this study, all fibula free flaps were harvested as osteocutaneous flaps, with the skin island serving as an external monitor of the buried flap’s perfusion, particularly the bone. The majority of studies available in the literature report fibula free flaps harvested as pure bone flaps without a skin component. These flaps, entirely buried without a visible viability monitor on the skin surface, did not provide a means for external perfusion monitoring. Notably, most studies did not perform any form of angiographic examination to confirm adequate perfusion of the biological reconstruction in the postoperative period. This lack of monitoring may explain the high rate of postoperative complications related to bone fragment fractures, which likely resulted from severe ischemia of the bone fragment and subsequent necrosis of the soft tissue component—conditions that could have been clinically silent.

**Figure 9:**
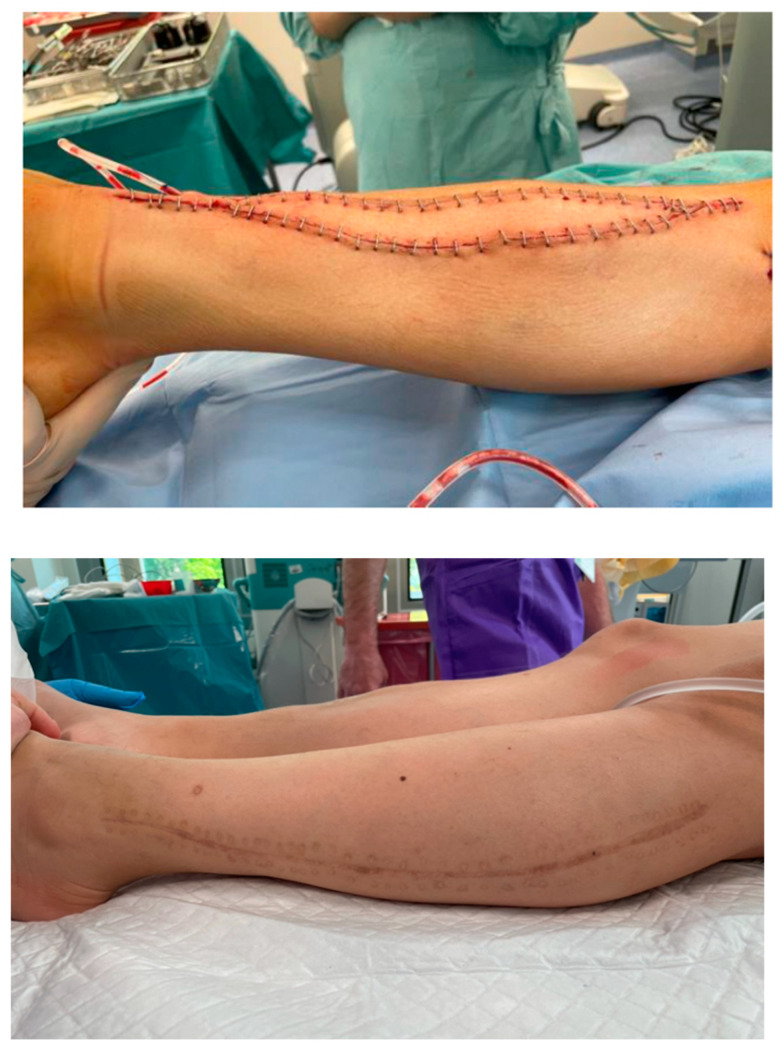
**Above:** An intraoperative photograph of a skin island’s inset within the treated lower leg—a healthy skin paddle without signs of vascular congestion. Attention is drawn to the tension-free closure of the skin layers. **Below:** A photograph of the fully healed donor site after harvesting a free fibula flap (13 months postoperatively). The donor site was closed primarily, without excessive tension, due to the small width of the harvested skin island.

In the analyzed study group, the proper appearance of the skin paddle served as an indirect indicator of the viability of the buried bony part of the flap (Figure 9). This is crucial because inadequate perfusion of the transferred fibula significantly impairs, and often prevents, the osseointegration of the flap with the adjacent bone stumps. Harvesting the skin island as a component of the FFF, despite the additional technical difficulty during dissection and the need to identify skin perforators, does not impose any additional burden on the patient. Simultaneously, it enables continuous and objective monitoring of the buried bone without the need for additional imaging tests, such as CT angiograms, which would expose the patient to radiation and contrast nephrotoxicity.

Furthermore, due to the small width of the harvested skin island, the donor site was closed primarily in each case, without the need for skin grafts (Figure 9). The skin paddle covering the vascularized bone graft also significantly reduced tension within the treated limb, contributing to the low incidence of wound healing disorders in the analyzed group of patients (9.5%). Given these benefits, it is difficult to argue against the routine harvesting of the free fibula flap as an osteocutaneous flap with an external ‘monitor’.

Both the appropriate technique of bone fixation and the delivery of well-perfused bone are crucial in achieving proper osseointegration, thereby mitigating the risk of postoperative fractures and pseudoarthrosis.

### 4.3. Double Microvascular Venous Anastomoses 

In microsurgery, the most common perioperative complication requiring urgent surgical intervention and frequently leading to partial tissue necrosis of the free flap is venous insufficiency, most commonly resulting from obstruction of the venous anastomosis [18]. Venous insufficiency is a much more common complication compared to arterial insufficiency regarding the free flaps. For this reason, in patients treated at the Institute of Mother and Child, in order to minimize the risk of the most common cause of flap perfusion impairment, two microsurgical venous anastomoses were performed during each procedure (Figure 6). One of them was performed in the deep venous system, the other in the superficial venous system.

The largest available meta-analysis, conducted by Shimbo et al., including almost 800 patients with free flap reconstructions within the lower limbs, showed that the incidence of venous insufficiency was significantly lower in the case of a flap-to-deep vein anastomosis than in the case of an anastomosis to the superficial vein (8.2% vs. 15.1%; *p* = 0.005) [19]. The rate of required reoperations after deep vein anastomosis was lower compared to superficial vein anastomosis, though this difference was not statistically significant (9.0% vs. 14.7%; *p* = 0.06). In our study group, only one flap (11%) exhibited symptoms of venous insufficiency localized within the skin island, such as skin cyanosis, accelerated capillary refill and congested blood flow upon puncture. Importantly, this condition affected only the internal microcirculation (specifically, the perforator supplying the skin island) and not the main vascular pedicle itself (the peroneal vessels). Consequently, a decision was made to excise the congested part of the flap (skin paddle) without the need for revising the microanastomosis, as adequate bone perfusion was confirmed.

### 4.4. Loading the Reconstructed Limbs and Late Postoperative Complications 

In this study, the average length of the defect (12.4 cm) was lower compared to the values reported in the literature (Jager et al.—17.5 cm, Ceruso et al.—15.8 cm) [7,20]. 

In contrast to cited articles reporting fracture rates in the range of 21–29%, no fractures were recorded in our analysis (Table 2). This satisfactory outcome was likely achieved through technical modifications of the procedure, including improvements in bone fixation techniques and ensuring adequate perfusion of the fibula during the initial healing period (utilizing a skin island for monitoring perfusion). Incorporating a bone allograft, initially serving as the primary reinforcement and shield for the fibula flap, reduces the risk of fractures within the reconstruction complex itself.

It is possible that due to less stress on the fibula, the hypertrophy of the free flap and its integration with the bone stumps occur more slowly. However, the enhanced mechanical strength from the outset allows pediatric patients to begin full weight-bearing sooner compared to reconstructions using the free fibula flap alone. Consequently, patients can return to daily activities earlier, reducing the risk of postoperative fractures.

While acknowledging our study’s relatively small sample size compared to others, our mean follow-up period exceeding 30 months and the observation that postoperative fractures mostly occurred early postoperatively provide promising results.

The lack of postoperative fractures in the study group may also be partly due to the location of the resected fragment within the bone—most of the reconstructions performed included the shaft only and were performed in the form of long diaphyseal intercalary grafts. Reconstructions including metaphyses are certainly more exposed to osseointegration impairment.

Despite the relatively low rate of complications observed in our analyzed group compared to other studies, such as that of Jager et al., particularly in terms of fractures, the approach presented in this study—extending the time to full weight-bearing of the limb (on average 12 months vs. 9.8 months in the literature)—appears justified. In our study, no postoperative fractures occurred. Although this longer period of restricted mobility may seem like a sacrifice for patients, it effectively prevented the need for surgical interventions necessitated by this complication.

We are also aware that a relatively short follow-up may falsely underestimate the long-term complications associated with resection and subsequent reconstruction in the tibial region. In the future, when we will be able to obtain information about the patients’ functional status in long-term follow-up, our research group wants to focus mainly on possible motor disorders and insufficiencies of the ankle and knee joints (e.g., ankle valgus). They undoubtedly constitute a serious problem after reconstructive treatment in the limb region.

In the field of limb reconstruction, a lot of attention is devoted to research on aspects such as modern bionic prostheses that can be controlled using the human mind, or assuming a completely different approach, i.e., advanced limb transplants from deceased donors, the survival of which together with the use of modern anti-rejection therapy may allow for long-term efficiency [21,22,23]. However, as we know, both of these paths have as many advantages as disadvantages [24]. The main aspect that should be considered when developing the “ideal” treatment method is also the financial aspect. The previously mentioned methods can cost amounts unattainable in many countries. The research team believes that the future of the treatments presented in the article is undoubtedly the possibility of obtaining a bone fragment used to strengthen the fibula flap not from a deceased donor, but from cell cultures using tissue bioengineering. We believe that such a bone fragment could be produced from bone cells on a specially prepared 3D matrix. The arrangement of cells would not be random but would correspond to the distribution of shear forces in the bone graft. It will be many years before we have a clear answer about the future of orthoplasty, but we hope that microsurgery will continue to play a key role.

## 5. Conclusions

The study group included in this analysis was relatively homogeneous in terms of patient characteristics, primarily consisting of pediatric patients in their teenage years most commonly affected by osteosarcoma and Ewing’s sarcoma.

The presented analysis demonstrates the effectiveness of this treatment approach—surgical resection combined with immediate reconstruction—which enables the avoidance of amputation following appropriate patient qualification, while achieving a satisfactory functional outcome. This approach effectively protects pediatric patients from disability at a young age, as evidenced by the preservation of all limbs in this study.

The obtained results of this analysis allow us to formulate the following conclusions:

1. Microvascular reconstructive surgery is an individually personalized and highly effective method of treating patients with primary bone tumors located in the limbs (after appropriate qualification) and provides satisfying functional outcomes.
2. Despite previous reports regarding the used reconstructive method available in the literature, technical modifications allowed for a significant reduction in the risk and even elimination of most local complications, in particular postoperative fractures and healing disorders as well as infections.
3. In the case of microvascular flaps transferred to reconstruct a bone defect, a skin island should be considered as a monitor of the viability of the buried part of the flap, which at the same time reduces the tension within the skin covering the reconstruction.
4. Despite the currently prevailing percentage of limb-sparing treatments, which in many clinical cases replace amputations, efforts should be made to increase the percentage of biological reconstructions, which help to avoid many complications typical for prostheses—mainly infections and damage to the endoprosthesis itself.
5. Appropriate qualification is an inherent element of microsurgical reconstruction procedures, as these tumors very often affect the joint area, where an endoprosthesis is in many cases an inevitable solution.

Further observation of included patients is an important aspect of future scientific research, mainly in terms of the possible occurrence of late orthopedic complications.

At the same time, the significant heterogeneity and variability of the nature of bone defects in patients with primary malignant bone tumors located in the limbs preclude the use of standardized treatment protocols in clinical practice.

## Figures and Tables

**Table 1:** Characteristics and treatment outcomes of the study group. OS—osteosarcoma; ES—Ewing’s Sarcoma; *—bearing 50% of the body weight on the treated lower limb.

No.	Sex	Age	Diagnosis	Bone Involved	Length of the Bone Defect	Follow-Up (Months)	Postoperative Complications	Local Recurrence/Distant Metastases	Partial Weight-Bearing * (Months)	Full Weight-Bearing (Months)	MSTS Scale Score (max. 30 Points)
1	M	11 y.o.	OS	Tibia	13 cm	51	Skin paddle necrosis	-	3	12	23
2	M	12 y.o.	ES	Tibia	10 cm	25	Wound dehiscence	-	2	10	27
3	M	15 y.o.	OS	Tibia	12 cm	34	none	-	3	12	29
4	F	14 y.o.	OS	Tibia	16 cm	27	none	Bone metastasis	3	14	30
5	M	16 y.o.	OS	Tibia	8 cm	32	none	-	2	10	24
6	F	15 y.o.	ES	Tibia	11 cm	25	none	-	3	12	28
7	M	12 y.o.	ES	Tibia	14 cm	23	none	-	4	12	27
8	F	13 y.o.	OS	Tibia	12 cm	31	none	Lung metastasis	3	12	28
9	M	13 y.o.	OS	Tibia	16 cm	29	none	-	4	14	29

**Table 2:** Comparison of the average length of the bone defect, time to initiation of full weight-bearing of the lower limb and the rate of postoperative fractures with other studies available in the literature.

Study	Average Length of the Bone Defect	Average Time to Initiation of Full Weight-Bearing	Number of Patients in Study Group	Percentage of Postoperative Fractures
Jager et al.	17.5 cm	6 months	7	29%
Ceruso et al.	15.8 cm	13.7 months	52	21%
Own study/Opyrchal et al.	12.4 cm	12 months	9	0%

## Data Availability

Data are contained within the article.
